# Factors Influencing Access to Health Services among Chronically Ill Older Adults with Physical Disabilities in the Era of the COVID-19 Outbreak

**DOI:** 10.3390/ijerph20010398

**Published:** 2022-12-27

**Authors:** Sutham Nanthamongkolchai, Athicha Tojeen, Korravarn Yodmai, Wanich Suksatan

**Affiliations:** 1 Department of Family Health, Faculty of Public Health, Mahidol University, Bangkok 10400, Thailand; 2 Faculty of Nursing, HRH Princess Chulabhorn College of Medical Science, Chulabhorn Royal Academy, Bangkok 10210, Thailand

**Keywords:** accessibility, COVID-19, health services, older adults, social support

## Abstract

Chronically ill older adults with physical disabilities frequently face difficulties in their daily lives and require essential health service access, especially in the COVID-19 context. This study aimed to examine the association between social support, perception of benefits due to disability and access to health services among chronically ill older adults with physical disabilities during this crisis in Thailand. A total of 276 chronically ill older adults with physical disabilities were included in this cross-sectional study. Self-reported questionnaires were assessed through multi-stage random sampling. Correlations between the independent variables and health service access were examined using multiple regression analysis. Of the respondents, 159 were female (59.6%). Most participants perceived benefits (58.8%) and access to health services (56.2%) at good levels, while social support was at a moderate level (47.9%). Stepwise multiple regression analysis showed that social support (β = 0.351), perception of benefits (β = 0.257) and age (β = 0.167) were positively correlated with health service access. The findings are relevant for health care providers and multi-professional teams, who should enhance older adults’ social support and perception of benefits to improve their access to health services, particularly among chronically ill older adults with physical disabilities, in the era of COVID-19.

## 1. Introduction

The coronavirus disease (COVID-19) outbreak has resulted in a significant global communicable disease burden. Starting in January 2020, the spread of the epidemic has steadily increased [1,2]; people have become infected, with the virus causing severe pneumonia and many deaths [3]. As of 14 October 2022, according to the World Health Organization (WHO), Thailand had reported 4,685,047 confirmed COVID-19 cases and 32,829 deaths, and 142,635,014 doses of the COVID-19 vaccine had been administered [4]. The COVID-19 pandemic has significantly impacted the lives of millions of people worldwide [5], especially chronically ill older adults with physical disabilities, who are most at risk from COVID-19 [6,7,8].

The National Statistical Office of Thailand reports that older adults with disabilities account for 20.6% of the nation’s elderly population [9], and older adults with physical disabilities outnumber other groups among elderly people [9]. Older adults with physical disabilities have limitations in caring for their health [10] and experience barriers to daily life, such as health care and access to government services; this can affect physical and mental health [11]. Although the welfare state assists some people with disabilities to live in society, welfare services are not very comprehensive according to a study conducted in Thailand [12,13]. Older adults with disabilities who had inadequate access to health services, especially to health care, may have barriers to accessing medical services in particular during the era of the COVID-19 pandemic [14,15].

Previous studies have examined access to health services among people with disabilities in various countries. For example, a study in South Africa found that a lower proportion of people with disabilities accessed health services compared to people without disabilities, and they also found that severe illness, education and age were associated with lower access to health services [16]. A study of older adults with disabilities before the COVID-19 pandemic in Peru reported that 65.4% of older adults with disabilities accessed health services through blood pressure testing for hypertension and blood testing for diabetes screening [17]. They also found that prior to the COVID-19 pandemic, a lower proportion of older persons with disabilities, especially those aged 65 and over, used health services compared to people below the age of 65 [17]. In Thailand, several studies have examined access to health services among people with disabilities prior to the COVID-19 pandemic. One study found that 88.5% of older adults with disabilities had easy access to medical care close to their homes and received good social support; the researchers also found that perceived factors regarding services provided for people with disabilities and social support were associated with access to health services [18]. The reasons for being unable to access health services are that traveling is inconvenient and that there are problems with expenses [10]. Previous studies reported that social support from their families and others was associated with access to health services and social welfare in older adults [19,20]. In addition, older adults who have lower access to health services might be unable to adopt health information seeking and scanning behaviors in order to improve their health behavior [20].

From the above, it can be seen that chronically ill older adults with physical disabilities have more problems and obstacles in accessing health services than other populations. These phenomena may have changed in Thailand during the COVID-19 pandemic. Chronically ill older adults with physical disabilities mostly do not have access to welfare benefits, mainly due to travel problems, ignorance of their rights and obstacles from their disabilities preventing them from accessing welfare [21]. They are also affected by a shortage of personnel, convenient locations and equipment to assist their rehabilitation [22]. Limited access to information, social support and participation in various social activities, including rehabilitation, are also factors affecting access to health services. A study on social support, perception of the benefits of disability and access to health services for chronically ill older adults with physical disabilities is unknown in Thailand, particularly in the era of the COVID-19 pandemic. The objective of this study was to determine the relationships between social support, perception of benefits of disability and access to health services for chronically ill older adults with physical disabilities. The results of this study could provide information to develop guidelines for enabling older adults with disabilities to access health services and have a good quality of life.

## 2. Materials and Methods

### 2.1. Study Design, Participants and Data Collection

This cross-sectional study used data from the “Factors Influencing the Quality of Life among Elderly People with Physical and Mobility Disabilities in Nakhonsawan Province” project. It was conducted between August and October 2020 during the COVID-19 pandemic through structured interview questionnaires. The sample size of 276 was calculated using the Daniel [23] formula: Z^2^ pq/e^2^, with a standard deviation (SD) of 0.47 (SD^2^ = 0.22), a 95% confidence level (Z = 1.96) and 5% accuracy (e = 0.05) from a previous study [24]. The participants in this study were selected using a multi-stage sampling technique from four districts (i.e., the Phaisalee, Phayuha Khiri, Banphot Phisai and Lat Yao districts) in Nakhon Sawan Province, Thailand. The sample size of this study was 276 (Phaisalee: *n* = 67, Phayuha Khiri: *n* = 67, Banphot Phisai: *n* = 67 and Lat Yao districts: *n* = 66). The inclusion criteria were (a) being aged 60 or over, (b) having a physical disability defined by the International Classification of Functioning, Disability and Health (ICF), which are defined as impairments in sensory functions, voice, speech, pain, or movement-related functions and neuro-musculoskeletal; or in nervous system structures, ear, eye, and related structures, speech, voice, or movement-related structures [25], and (c) having been diagnosed by a physician with one or more chronic illnesses (i.e., hypertension, diabetes, chronic obstructive pulmonary disease, cardiovascular and heart disease, and cancer). We excluded participants who were unwilling to participate in this study.

### 2.2. Dependent Variable

#### Access to Health Services

We used the Access to Health Service Scale (AHSS) to assess access to health services. This questionnaire was developed by Nanthamongkolchai et al. [13]; it includes 16 items with five answer options that are scored between 1.00 and 5.00 points. A score higher than 80% of the total indicates high access to health services [13]. The Cronbach’s alpha coefficient in this study was 0.95.

### 2.3. Independent Variable

#### 2.3.1. Social Support

We used the Social Support Scale (SSS) to assess perceived social support among the participants. The SSS, which was developed by Nanthamongkolchai et al. [13], consists of 25 items about social support evaluated through responses that concern emotions, recognition and appreciation, participation, instruments and information support. The total social support scores are classified into three levels: ≤59 = low, 60–79 = moderate and ≥80 = high. Higher scores indicate a high level of social support [13]. The Cronbach’s alpha coefficient in this study was 0.95.

#### 2.3.2. Perception of the Benefits of Disability

The Perception of the Benefits of Disability Scale (PBDS) was developed by Nanthamongkolchai et al. [13] to assess the perceived benefits of disability. The PBDS comprises 16 items with 2 options scored as 0 or 1. The scores are classified into three levels: ≤59 = low, 60–79 = moderate and ≥80 = high. Higher scores indicate a high level of perception of the benefits of disability [13]. The Cronbach’s alpha coefficient in this study was 0.73.

#### 2.3.3. Background Information

Six open-ended or multiple-choice questions in the demographic questionnaire were about the participants’ sex, age, education, marital status, income and the number of years of disability. The answers were used to assess the respondents.

### 2.4. Data Analysis

The Statistical Package for the Social Sciences (SPSS) version 24 (IBM Corp., Armonk, NY, USA) was used to analyze the data. The data are presented as frequencies, percentages (%), means (M) and standard deviations (SD). Stepwise multiple regression analysis was used to determine the associated factors of the participants’ access to health services. *p* < 0.05 was considered significant.

## 3. Results

### 3.1. Participant Characteristics

Table 1 shows the study participants’ summary statistics. Of the participants, 59.6% were female (*n* = 159). The average age was 74.0 years (range 60–89 years), and 97.0% had a primary education (*n* = 259). Most participants had physical disabilities with reduction defects of unspecified limb (*n* = 105, 39.3%). Most participants (92.5%) had a monthly income of less than USD 142 (*n* = 247); 56.2% were married; and the average number of years of disability was 13.2 years.

#### 3.1.1. Social Support, Perception of Benefits of Disability and Access to Health Services

**Figure 1:**
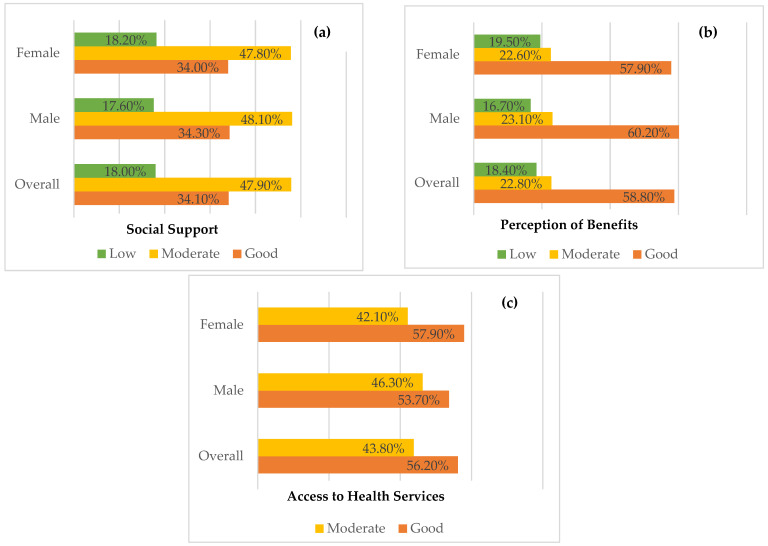
Participant levels of (**a**) social support; (**b**) perception of benefits; and (**c**) access to health services.

As shown in Figure 1a, the overall social support of the participants was at a moderate level (47.9%, *n* = 128). When considered by sex, female participants (47.80%, *n* = 76) and male participants (48.10%, *n* = 52) had a moderate level. Moreover, the overall perception of the benefits of disability of the participants was at a good level (58.80%, *n* = 157). When considered by sex, female participants (57.90%, *n* = 92) and male participants (60.20%, *n* = 65) had a good level (Figure 1b). In addition, the overall access to health services of the participants was at a good level (56.20%, *n* = 150). When considered by sex, female participants (57.90%, *n* = 92) and male participants (53.7%, *n* = 58) had a good level, as shown in Figure 1c.

#### 3.1.2. Factors Associated with Access to Health Services

Multiple regression analyses were performed to examine the potential factors associated with access to health service in the study population (Table 2). Three predictors were discovered: social support, perception of benefits and age (β = 0.351, 0.257, and 0.167, respectively); these factors predicted the participants’ access to health services with an accuracy of 23.3%.

## 4. Discussion

In this study, we examined relationships between participant demographic characteristics and other social factors to explore how they impacted access to health services. We discovered that the three independent variables in this study could predict access to health services among chronically ill older adults with physical disabilities, accounting for approximately 23.3% of the variance.

In this study, 56.2% of the participants had a high level of access to health services, while 43.8% had a moderate level. Almost half (47.9%) of the participants had a moderate level of social support, while 34.1% had a good level of social support. The results of this study are consistent with previous studies that found that older adults with disabilities had moderate- and high-level access to health services [26,27]. However, compared to chronically ill older adults without a disability, the proportion was lower for older adults with disabilities [26,28,29]. This can be explained by the fact that chronically ill older adults with physical disabilities have less access to health services, which may partly be due to travel difficulties due to disabilities, especially during the COVID-19 pandemic. Interestingly, our study found that 65.9% of the participants had moderate to low social support during the COVID-19 pandemic. A previous study of older adults in Thailand during the pandemic revealed that only 34.1% of the participants had a good level of social support, which indicates that the COVID-19 epidemic contributed to a reduction in access to health services and social support for older adults [30]. Previous studies also demonstrated that older adults who had high perceived social support from their families and social embeddedness were associated with good access to health services and social welfare in older adults [19,20].

The analysis of factors related to and predictive of access to health services among chronically ill older adults with physical disabilities found that social support, perceived benefits and age were statistically correlated and could predict access to health services. This result is consistent with previous studies that found that social support and perceived benefits are associated with access to health services for elderly people with disabilities [31,32]. Social support is a key variable in describing older adults’ access to health services. This may be because, even during the COVID-19 pandemic, older people with disabilities have been encouraged to receive support from their family and relatives, although it decreased the past [13]. In addition, a previous study in China revealed that Chinese older adults who have lower access to health services might be unable to adopt health information seeking and scanning behaviors in order to improve their health behavior, especially in older adults living in rural area [20].

The perceived benefit factor was related to access to health services: 58.8% of the participants perceived their benefits to be at a high level. In particular, they could receive public services anywhere when they were sick (86.5%), receive health services from a health facility near their home (92.1%) and seek advice on health problems from a health facility close to home (95.9%). This shows that, when older adults are more aware of their benefits, they can access health services, even during the COVID-19 epidemic. The results of this study are consistent with the studies by Malone et al. [33] and Mulumba et al. [34], which found that perceived benefits were associated with access to health services among older adults with disabilities.

In this study, the last predictor of access to health services among chronically ill older adults with physical disabilities was age. We found that chronically ill older adults with physical disabilities had greater access to health services. This can be explained by the fact that older adults, when ill, were likelier to receive support from their families due to fears about the impact of the COVID-19 pandemic. Therefore, they were able to receive services in health care facilities. The results of this study differ from those of previous studies, which found that older people with disabilities have less access to health services [16,17].

This study’s results indicate that social support and perceived benefits were associated with older adults’ access to health services during the COVID-19 pandemic. The findings show that support from family and society in terms of health information, emotional support, material support and participation in activities is essential [35,36], including acknowledging the benefits for older adults with disabilities [33]. In addition, government policy needs to account for the need of older adults to rely on family/social support systems to preserve access, particularly in older adults with physical disabilities who have impairments, activity limitations and participation restrictions.

The strength of our study was that it was the first study in Thailand during the COVID-19 pandemic that involved chronically ill older adults with physical disabilities, most of whom were affected by this situation. We investigated the influence of the participants’ characteristics, perceptions of benefits and social support on access to health services, which might help the future situation of this population. However, our study had some limitations. First, the cross-sectional survey conducted in this study could not reach the same conclusions as experimental or long-term studies. Future studies should be able to draw causal inferences in a longitudinal prospective study, which is needed to investigate the direction and cause of each factor affecting the access to health services of chronically ill older adults with physical disabilities. Second, our sample size was small due to issues with one province in Thailand. Future studies are needed with large sample sizes and multiple settings in other urban and rural residentials, or provinces, and nations to identify the factors influencing access to health services among this population. Third, our findings might not have been generalizable to chronically ill older adults with physical disabilities at home during the COVID-19 outbreak in Thailand because we recruited participants in only one province in Thailand, which could indicate selection bias. Finally, we examined the relationships among the participant characteristics, social support, perceptions of benefits, and access to health services, and only 23.3% of the variance could predict access to health services among this population. Future studies should include other essential factors, including social isolation, eHealth and mHealth literacy, and environmental and cultural factors, to identify factors associated with access to health services among chronically ill older adults with physical disabilities.

## 5. Conclusions

This study revealed that chronically ill older adults with physical disabilities during the COVID-19 outbreak in one province in Thailand had a high level of access to health services. We also found that social support, perceptions of benefits and age were related to access to health services among this population. Based on this study, policymakers should factor in social support and perception of benefit into designing interventions to increase access to health care services and improve health care outcomes for the aging population of Thailand especially for those with chronic illness and disabilities.

## Figures and Tables

**Table 1:** Participant characteristics.

Factor	N	%
Sex		
Male	108	40.4
Female	159	59.6
Age		
60–69	105	39.3
70–79	95	35.6
80–89	67	25.1
Mean = 74.0, SD = 8.00, Max = 89, Min = 60		
Education level		
Primary	259	97.0
Secondary	8	3.0
Family income (USD)		
≤142	247	92.5
143–285	17	6.4
≥286	3	1.1
Mean = 71.96, SD = 67.77, Max = 572, Min = 40		
Marital status		
Married	150	56.2
Widowed/Divorced	98	36.7
Single	19	7.1
Type of Physical Disabilities		
Reduction defects of upper limb	36	13.5
Reduction defects of lower limb	99	37.1
Reduction defects of unspecified limb	105	39.3
More than 1 physical disability and other	27	10.1
Number of years disabled		
≤10	164	61.4
11–20	66	24.7
21–30	15	5.6
≥31	22	8.2
Mean = 13.34, SD = 13.20, Min = 1, Max = 70		

**Table 2:** Factors associated with access to health services.

Predictive Factor	B	β	t	*p*-Value
Age	0.146	0.167	2.962	0.003
Income	−3.918	−0.013	−0.242	0.809
Number of years disabled	0.019	0.038	0.670	0.504
Social support	0.141	0.351	6.317	<0.001
Perception of benefits	0.546	0.257	4.597	<0.001

Notes: Constant = 30.749, R^2^ = 0.233, Adj R^2^ = 0.218.

## Data Availability

Not applicable.
